# Antecedent causes of a measles resurgence in the Democratic Republic of the Congo

**DOI:** 10.11604/pamj.2015.21.30.6335

**Published:** 2015-05-15

**Authors:** Heather Melissa Scobie, Benoît Kebela Ilunga, Audry Mulumba, Calixte Shidi, Tiekoura Coulibaly, Ricardo Obama, Jean-Jacques Muyembe Tamfum, Elisabeth Pukuta Simbu, Sheilagh Brigitte Smit, Balcha Masresha, Robert Tyrrell Perry, Mary Margaret Alleman, Katrina Kretsinger, James Goodson

**Affiliations:** 1 Centers for Disease Control and Prevention, Atlanta, Georgia, USA; 2 Epidemic Intelligence Service, Centers for Disease Control and Prevention, Atlanta; 3 Ministry of Public Health, Kinshasa, Democratic Republic of the Congo; 4 World Health Organization, Kinshasa; 5 National Institute for Biomedical Research, Kinshasa; 6 National Institute for Communicable Diseases, Johannesburg, South Africa; 7 World Health Organization African Regional Office, Brazzaville, Republic of the Congo; 8 World Health Organization, Geneva, Switzerland

**Keywords:** Measles, outbreak, elimination, immunization, vaccination, surveillance, DRC, RDC

## Abstract

**Introduction:**

Despite accelerated measles control efforts, a massive measles resurgence occurred in the Democratic Republic of the Congo (DRC) starting in mid-2010, prompting an investigation into likely causes.

**Methods:**

We conducted a descriptive epidemiological analysis using measles immunization and surveillance data to understand the causes of the measles resurgence and to develop recommendations for elimination efforts in DRC.

**Results:**

During 2004-2012, performance indicator targets for case-based surveillance and routine measles vaccination were not met. Estimated coverage with the routine first dose of measles-containing vaccine (MCV1) increased from 57% to 73%. Phased supplementary immunization activities (SIAs) were conducted starting in 2002, in some cases with sub-optimal coverage (≤95%). In 2010, SIAs in five of 11 provinces were not implemented as planned, resulting in a prolonged interval between SIAs, and a missed birth cohort in one province. During July 1, 2010-December 30, 2012, high measles attack rates (>100 cases per 100,000 population) occurred in provinces that had estimated MCV1 coverage lower than the national estimate and did not implement planned 2010 SIAs. The majority of confirmed case-patients were aged <10 years (87%) and unvaccinated or with unknown vaccination status (75%). Surveillance detected two genotype B3 and one genotype B2 measles virus strains that were previously identified in the region.

**Conclusion:**

The resurgence was likely caused by an accumulation of unvaccinated, measles-susceptible children due to low MCV1 coverage and suboptimal SIA implementation. To achieve the regional goal of measles elimination by 2020, efforts are needed in DRC to improve case-based surveillance and increase two-dose measles vaccination coverage through routine services and SIAs.

## Introduction

Measles is a highly-infectious and potentially fatal viral disease characterized by fever and rash. In 2012, measles caused an estimated 122,000 vaccine-preventable deaths worldwide [1]. Estimated measles vaccine effectiveness (VE) is 84% when administered at 9-11 months of age, and 93% when given at ≥12 months of age [2]. Because >93%-95% population immunity is necessary to prevent measles epidemics, the World Health Organization (WHO) recommends all children receive two doses of measles vaccine [2, 3].

In 2008, countries in the WHO African Region (AFR) adopted a measles pre-elimination goal to be achieved by the end of 2012 with the following targets: 1) >98% reduction in estimated regional measles mortality compared with 2000; 2) national measles incidence of <5 cases per 1,000,000 population per year; 3) >90% national coverage with the first dose of measles-containing vaccine (MCV1) and >80% MCV1 coverage in all districts; and 4) >95% coverage in all districts for MCV supplementary immunization activities (SIAs). Also included were surveillance performance targets of: 1) ≥2 cases of non-measles febrile rash illness per 100,000 population; 2) ≥1 suspected measles case investigated with blood specimens in ≥80% of districts [4].

In the Democratic Republic of the Congo (DRC), MCV1 given at 9 months of age was introduced into the Expanded Programme on Immunization in 1977 [5]. The Reaching Every District (RED) approach to strengthening RI services was implemented beginning in 2003 [6]. To provide a second opportunity for measles vaccination, nationwide measles SIAs started in 2002, using a phased approach intended to cover the country every 3 years [7]. Integrated Disease Surveillance and Response (IDSR) was established in 2000 with aggregate reporting of 18 infectious diseases, including measles. Case-based surveillance with laboratory confirmation of suspected measles cases was established in 2003.

In 2011, AFR countries adopted a goal for measles elimination by 2020 [8]. However, starting in 2010 and continuing through 2014, DRC experienced the largest nationwide measles outbreak in AFR since the start of accelerated measles control efforts [9–12]. DRC is a key country for regional elimination efforts because of its large population, central location with nine international borders, and persistent reservoir of circulating measles viruses [9, 13]. We conducted a descriptive epidemiological analysis using immunization and surveillance data to understand the likely causes of the measles resurgence and develop recommendations for elimination efforts.

## Methods

### Study setting

In 2012, DRC had an estimated population of 78.1 million persons, 3.1 million live births, and 14.7 million children aged <5 years [14]. The DRC health system has 513 zones de santé (health districts) in 11 provinces. Challenges for vaccination include a large target population, poorly accessible areas, complex logistics, a lack of government funding, weak health systems, civil unrest, and displaced persons [15–17]. The number of displaced persons peaked in 2013, with 2.9 million internally displaced persons (IDPs) in the eastern and northern provinces of DRC and 438,869 Congolese in other African countries [18–21]. The last population census was conducted in 1981 making estimation of the vaccination target population challenging.

### Measles immunization, 2002-2012

To assess measles vaccination coverage in comparison with AFR indicator targets, we calculated MCV1 administrative coverage for 2004-2012 at the national, provincial, and district levels by dividing the reported number of MCV1 doses administered by the number of targeted children aged <12 months, according to the DRC Ministry of Public Health (MOPH), multiplied by 100 (national target: >90%; district target: >80%). The number of districts reporting >80% MCV1 coverage was divided by the number of districts expected to report and multiplied by 100 (target: 100%) [22]. We reviewed WHO-United Nations Children's Fund (UNICEF) annual estimates of national MCV1 coverage among children aged <12 months, and population-based survey estimates of national and provincial MCV1 coverage among children aged 12-23 months during 2004-2012 [23–27]. We reviewed implementation dates, administrative coverage, and survey results from SIAs and outbreak response immunization (ORI) during 2002-2012; the AFR target of >95% SIA coverage in all districts could not be assessed due to unavailable data [22].

### Measles surveillance, 2004-2012

Through IDSR, aggregate numbers of suspected measles cases and deaths were reported weekly from districts to the national level [28]. Through measles case-based surveillance, suspected measles cases were investigated using a case investigation form and laboratory testing of a blood specimen at the National Institute for Biomedical Research in Kinshasa [29]. Specimens were tested using standard enzyme-linked immunosorbent assays for measles-specific immunoglobulin M (IgM) antibodies; specimens with a negative or indeterminate result were tested for rubella-specific IgM (Enzygnost ELISA^™^, Siemens Healthcare Diagnostics Products, Marburg, Germany). A suspected measles case was defined as an illness with maculopapular rash and fever and one or more symptoms of cough, coryza, or conjunctivitis, or where a clinician suspects measles. A laboratory-confirmed measles case was defined as a suspected measles case with a positive laboratory test result for measles-specific IgM in the absence of measles vaccination within 30 days of specimen collection. An epidemiologically-linked case was defined as a suspected measles case having contact (or living in the same district) with a laboratory-confirmed measles case whose rash onset was within the preceding 30 days. A clinically-compatible case was defined as a suspected measles case without a laboratory test result or established epidemiological link. We classified all laboratory-confirmed, epidemiologically-linked, and clinically-compatible measles cases as confirmed measles cases.

We analyzed de-duplicated IDSR and measles case-based surveillance data from 2004-2012. To assess case-based surveillance performance in comparison with AFR targets, we calculated two indicators as follows:1) the number of districts annually reporting ≥1 suspected measles case with a collected blood specimen divided by the number of districts expected to report (target: ≥80%); and 2) the number of suspected measles cases reported annually and “discarded” as non-measles because of a negative test result divided by the estimated population from DRC MOPH, multiplied by 100,000 (target: ≥2.0). Estimated annual measles incidence was calculated by dividing the number of suspected cases reported through IDSR by the estimated annual population from DRC MOPH, multiplied by 1,000,000 (target: <5) [22].

### Epidemiological description of the measles resurgence

We conducted an epidemiological analysis of the resurgence period, defined as starting in the first month with a confirmed measles outbreak in 2010 and continuing through the end of 2012, using IDSR and case-based surveillance data from July 1, 2010-December 31, 2012. A confirmed measles outbreak was defined as ≥3 laboratory-confirmed cases occurring in a district in one month. We analyzed suspected measles cases and deaths reported through IDSR by date of rash onset, province, and district. Confirmed measles cases reported through case-based surveillance were analyzed by date of rash onset, age, vaccination status, province, and district. Cumulative measles attack rates were calculated by dividing the number of suspected cases reported through IDSR during the resurgence period, or a 6-month subset of the resurgence period, by the estimated 2012 population from DRC MOPH and multiplying by 100,000. Cumulative measles attack rates and confirmed measles outbreaks by district for 6-month periods were mapped. Analyses were conducted using Excel (Microsoft Corporation), SAS version 9.3 (SAS Institute), and ArcGIS (ESRI).

To identify circulating measles virus strains, nasopharyngeal swabs were collected and tested at the National Institute for Communicable Diseases in South Africa. Ribonucleic acid (RNA) was extracted using the QIAamp^®^ viral RNA mini kit (QIAGEN^®^) and amplified by reverse-transcriptase polymerase chain reaction. Amplicons were sequenced and analyzed using Sequencher software (Gene Codes Corporation 4.1.4, Ann Arbor, MI). Phylogenetic analysis of the viral nucleoprotein (N) gene relative to WHO measles reference strains was performed using the maximum likelihood algorithm of MEGA version 5.1 software with bootstrap test of phylogeny [30].

## Results

### Measles immunization, 2002-2012

National MCV1 administrative coverage among children aged <12 months increased from 64% to 89%, and WHO-UNICEF estimates of MCV1 coverage increased from 57% to 73% during 2004-2012. National MCV1 coverage among children aged 12-23 months estimated from surveys was 63% in 2007, 67% in 2010, and 75% in 2012. MCV1 coverage survey estimates were lower than reported administrative coverage for comparable years. In all years, MCV1 administrative coverage varied substantially by province and was below the target of >90% at the national level and >80% in all districts (Table 1).

**Table 1:** Coverage with the routine first dose of measles-containing vaccine by administrative reporting, survey, and WHO-UNICEF estimate during 2004–2012 and cumulative measles attack rates during July 1, 2010–December 31, 2012, Democratic Republic of the Congo

Province	MCV1 administrative coverage (%)^a^									MCV1 coverage (%) by survey^b^			Cumulative attack rate^c^
	2004	2005	2006	2007	2008	2009	2010	2011	2012	DHS 2007	MICS 2010	EPI 2012	
Katanga	66	76	79	74	83	88	91	89	91	52	66	72	774
Maniema	48	81	69	85	82	87	92	96	97	45	46	67	543
Kasaï Oriental	63	65	68	75	73	85	86	88	89	59	52	62	504
Province Orientale	45	50	53	70	71	73	78	70	75	50	60	62	366
Equateur	51	56	74	83	74	86	80	83	84	42	51	74	112
Sud Kivu	50	63	76	104	84	87	94	93	96	84	57	86	107
Bandundu	75	73	77	89	71	88	90	88	91	79	91	88	95
Kasaï Occidental	65	73	73	71	82	94	93	80	104	45	49	54	68
Bas-Congo	79	84	82	88	83	91	89	78	85	88	77	78	46
Kinshasa	78	80	77	76	77	86	85	79	82	91	86	88	25
Nord Kivu	86	95	81	80	78	91	93	98	94	86	80	88	13
National	64	70	73	79	77	86	87	85	89	63	67	75	270
National WHO-UNICEF estimates^d^	57	61	63	68	64	72	74	74	73	-	-	-	-
% of districts with MCV1 >80%^e^	26	35	37	49	44	68	68	60	68	-	-	-	-

Abbreviations: MCV1=first dose of measles-containing vaccine, DHS=Demographic Health Survey, MICS=Multiple Indicator Cluster Survey, EPI=Expanded Programme on Immunization, Democratic Republic of the Congo, WHO=World Health Organization, UNICEF=United Nations Children's Fund, IDSR=Integrated Disease Surveillance and Response, MOPH=Ministry of Public Health

a Calculated by dividing the number of MCV1 doses given by the targeted number of children <12 months of age and multiplying by 100; target >90%

b Estimates from coverage surveys conducted among children aged 12–23 months of age [24–26]

c Calculated by dividing the number of suspected cases reported to IDSR during July 1, 2010 to December 31, 2012 by the estimated 2012 population from the MOPH and multiplying by 100; target >90%

d Annual estimates of national MCV1 coverage for children <12 months of age [23]

e Calculated by dividing the number of districts reporting >80% MCV1 coverage by the number of districts expected to report and multiplying by 100; target: 100%

**Figure 1:**
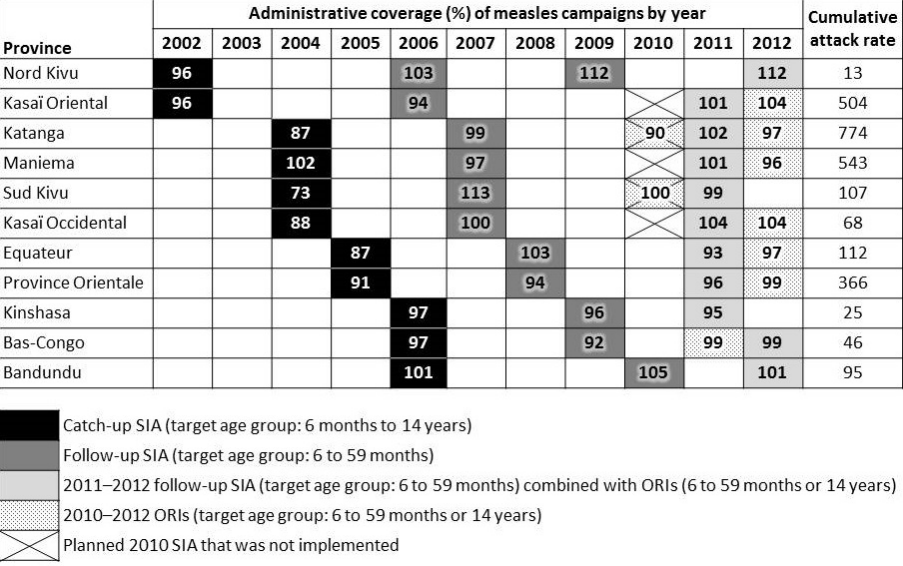
Administrative measles vaccination coverage from supplementary immunization activities (SIA) and outbreak response immunization (ORI) by province and year during 2002-2012, and cumulative measles attack rates reported through Integrated Disease Surveillance and Response during July 1, 2010-December 31, 2012, Democratic Republic of the Congo

A phased catch-up SIA targeting children aged 6 months-14 years was implemented during 2002-2006 (median reported administrative coverage by province: 96%; range: 73%-102%), and a phased follow-up SIA targeting children aged 6-59 months was completed during 2006-2010 (median reported administrative coverage: 99%; range: 92%-113%). A 4-year gap between these SIAs occurred in three provinces (Nord Kivu, Kasaï Oriental, and Bandundu). In 2009, another phased follow-up SIA was initiated in Nord Kivu (reported administrative coverage: 112%); however, the phase planned for 2010 was not implemented, leading to a 4-5 year gap between SIAs in five provinces (Kasaï Oriental, Kasaï Occidental, Katanga, Maniema, and Sud Kivu) and a missed cohort of children born in 2006 in Kasaï Oriental (Figure 1 and Table 2). After the start of the 2010 outbreaks, the entire country was covered by a combination of subnational measles follow-up SIAs and ORIs targeting children aged 6-59 months or 6 months-14 years during 2011-2012 (median reported administrative coverage: 101%; range: 93%-112%) (Figure 1 and Table 3).

**Table 2:** Coverage from measles supplementary immunization activities by administrative reporting and post-campaign surveys, Democratic Republic of the Congo, 2002–2010

Province	Year	Month	SIA type^a^	Target age group	Children vaccinated	Administrative coverage (%)^b^	Coverage by survey (%)^c^
Kasaï Oriental	2002	Dec.	Catch-up	6m–14y	3,478,261	96	-
Nord Kivu	2002	Dec.	Catch-up	6m–14y	2,076,563	96	-
Kasaï Occidental	2004	Oct.–Nov.	Catch-up	6m–14y	2,473,668	88	-
Katanga	2004	Oct.–Nov.	Catch-up	6m–14y	3,784,714	87	-
Maniema	2004	Oct.–Nov.	Catch-up	6m–14y	768,987	102	-
Sud Kivu	2004	Oct.–Nov.	Catch-up	6m–14y	1,577,385	73	-
Equateur	2005	Oct.	Catch-up	6m–14y	3,268,004	87	-
Province Orientale	2005	Oct.	Catch-up	6m–14y	3,691,667	91	-
Bas-Congo	2006	Dec.	Catch-up	6m–14y	1,246,371	97	-
Kinshasa	2006	Nov.	Catch-up	6m–14y	2,659,229	97	98^d^
Bandundu	2006	Nov.	Catch-up	6m–14y	3,064,629	101	
Nord Kivu	2006	Oct.	Follow-up	6–59m	900,330	103	
Kasaï Oriental	2006	May	Follow-up	6–59m	1,257,999	94	-
Kasaï Occidental	2007	Aug.–Sep.	Follow-up	6–59m	1,068,950	100	94^d^
Katanga	2007	Aug.–Sep.	Follow-up	6–59m	1,624,002	99	
Maniema	2007	Sep.	Follow-up	6–59m	290,803	97	
Sud-Kivu	2007	Sep.	Follow-up	6–59m	821,937	113	-
Equateur	2008	Nov.	Follow-up	6–59m	1,449,659	103	-
Province Orientale	2008	Nov.	Follow-up	6–59m	1,361,433	94	-
Bas-Congo	2009	Nov.	Follow-up	6–59m	482,070	92	-
Kinshasa	2009	Nov.	Follow-up	6–59m	1,086,047	96	-
Nord Kivu	2009	Nov.	Follow-up	6–59m	1,073,437	112	-
Bandundu	2010	Jan.	Follow-up	6–59m	1,274,163	105	-

Abbreviations: m=month, y=year, SIA=Supplementary Immunization Activity

a “Catch-up” SIAs are one-time campaigns targeting a wide age range with the aim to quickly reduce measles susceptibility in the population. “Follow-up” SIAs are periodic campaigns targeting children born since the last SIA to reduce the accumulation of susceptible children

b Calculated by dividing the number of measles vaccine doses administered by the targeted number of children and multiplying by 100; target >95%

c Estimates from coverage surveys conducted among children in the age group targeted

d 2006 and 2007 survey coverage estimates are overall estimates for the three campaigns indicated

**Table 3:** Coverage from measles supplementary immunization activities during 2011–2012 and outbreak response immunization during 2010–2012 by administrative reporting and post-campaign surveys, Democratic Republic of the Congo

Province	Districts in province	Districts covered	Year	Month	SIA type/ ORI^a^	Target age group	Children vaccinated	Administrative coverage (%)^b^	Coverage by survey (%)^c^
Bandundu	52	46	2012	Jan.	Follow-up	6–59m	1,239,803	99	-
		6	2012	Jan.	ORI	6–59m	129,000	102	-
		5	2012	Oct.–Nov.	ORI	6m–14y	507,034	105	-
Bas-Congo	31	5	2011	Jul.	ORI	6–59m	65,657	99	-
		18	2012	Jan.	Follow-up	6–59m	303,786	97	-
		9	2012	Jan.	ORI	6m–14y	530,149	100	-
		1	2012	Oct.	ORI	6m–14y	82,842	102	-
Equateur	69	3	2011	Jul.	ORI	6–59m	106,103	91	-
		66	2011	Sep.	Follow-up	6–59m	1,419,411	94	-
		1	2012	Apr.–May	ORI	6m–14y	128,752	108	-
		6	2012	Sep.	ORI	6m–14y	477,066	94	96
Kasaï Occidental	44	1	2011	Jan.–Feb.	ORI	6m–14y	123,045	93	-
		3	2011	Mar.–Apr.	ORI	6m–14y	430,571	104	-
		9	2011	May	ORI	6m–14y	792,389	111	-
		4	2011	Jul.	ORI	6–59m	125,109	106	-
		29	2011	Jul.	Follow-up	6–59m	684,176	98	-
		5	2012	Aug.	ORI	6m–14y	348,176	104	94
Kasaï Oriental	51	31	2011	Jul.	Follow-up	6–59m	729,336	103	-
		4	2011	Mar.–Apr.	ORI	6–59m	159,542	104	-
		14	2011	Apr.	ORI	6–59m	564,252	99	-
		2	2011	Jun.	ORI	6m–14y	268,586	99	-
		8	2012	Mar.	ORI	6m–14y	478,588	104	-
		1	2012	Nov.	ORI	6–59m	67,152	103	-
Katanga	68	2	2010	Oct.–Dec.	ORI	6m–14y	144,071	90	-
		27	2011	Jan.–Aug.	ORI	6m–14y	1,847,096	102	d
		24	2011	May	ORI	6m–14y	1,917,060	103	-
		17	2011	Jul.	Follow-up	6–59m	585,633	105	-
		2	2011	Oct.–Dec.	ORI	6m–14y	167,485	74	-
		10	2012	Aug.	ORI	6m–14y	941,879	97	68
Kinshasa	35	25	2011	Dec.	Follow-up	6–59m	1,089,048	101	-
		10	2011	Dec.	ORI	6m–14y	937,439	89	-
Maniema	18	14	2011	May	ORI	6m–14y	833,512	101	95
		4	2011	Jul.	Follow-up	6–59m	58,202	103	98
		2	2012	Aug.	ORI	6m–14y	81,247	96	94
Nord Kivu	28	19	2012	Jan.	Follow-up	6–59m	948,237	112	-
		5	2012	Jan.	ORI	6–59m	284,592	107	-
		1	2012	Aug.	ORI	6m–14y	197,820	116	-
Province Orientale	83	7	2011	Jul.	ORI	6–59m	119,586	93	-
		1	2011	Sep.	ORI	6m–14y	74,549	108	-
		76	2011	Sep.	Follow-up	6–59m	1,456,498	96	-
		2	2012	Aug.–Nov.	ORI	6m-14y	97,332	99	-
Sud Kivu	34	1	2010	Nov.–Dec.	ORI	6m–14y	88,853	100	-
		4	2011	Feb.–Jun.	ORI	6m–14y	250,877	81	-
		25	2011	May	ORI	6m–14y	1,826,722	102	-
		4	2011	Jul.	Follow-up	6–59m	95,955	105	-

Abbreviations: m=month, y=year, SIA=Supplementary Immunization Activity, ORI=Outbreak Response Immunization

a “Follow-up” SIAs are periodic campaigns targeting children born since the last SIA to reduce the accumulation of susceptible children ORIs are campaigns conducted in response to confirmed outbreaks, with target age and geographic area based on epidemiology and available resources

b Calculated by dividing the number of measles vaccine doses administered by the targeted number of children and multiplying by 100; target >95%

c Estimates from coverage surveys conducted among children in the age group targeted

d District-level coverage survey estimates available for campaigns conducted by MSF in 19 of 27 districts [12]

During 2002-2012, ≤95% administrative coverage was reported from ≥1 measles SIA in eight of 11 provinces (Figure 1). Availability of SIA coverage estimates from surveys during this period was limited. Coverage survey estimates for SIAs implemented in 2006, 2007 and 2011 were >95% in Kinshasa, Bandundu, Nord Kivu, and Maniema, and 94% in Kasaï Occidental, Katanga, and Maniema (Table 2, Table 3). Coverage survey estimates for ORIs implemented during 2010-2012 were >95% in Equateur, 92%-95% in Kasaï Occidental and two ORIs in Maniema, and 68% in Katanga (Table 3). In general, SIA and ORI coverage survey estimates were lower than reported administrative coverage (Table 2, Table 3).

### Measles surveillance, 2004-2012

Measles case-based surveillance did not meet both annual performance indicator targets during 2004-2012. The number of suspected measles cases reported through case-based surveillance (16,789) was 3.6% of the total reported to IDSR (459,326); the number of suspected measles deaths reported through case-based surveillance (52) was 0.6% of the total reported to IDSR (8,400). Suspected measles incidence reported through IDSR did not meet the target of <5 per 1,000,000 population during 2004-2012. During 2006-2009, incidence decreased 96%, from 1,164 to 47 per 1,000,000. During 2010-2011, incidence increased from 73 to 1,769 per 1,000,000, and was 935 per 1,000,000 in 2012 (Table 4).

**Table 4:** Measles surveillance data reported through Integrated Disease Surveillance and Response and measles case-based surveillance, Democratic Republic of the Congo, 2004–2012

	Year									
Indicator	2004	2005	2006	2007	2008	2009	2010	2011	2012	Total
***IDSR***										
No. suspected measles cases	42,827	53,022	79,873	55,440	12,490	3,364	5,365	133,907	73,038	459,326
No. suspected measles deaths	1,097	595	1,181	1,463	247	73	82	1,650	2,012	8,400
Suspected measles incidence per 1,000,000^a^	640	782	1,164	823	180	47	73	1,769	935	6,412
***Case-based surveillance***										
No. suspected measles cases	769	1,364	2,148	1,923	1,178	701	1,453	3,170	4,083	16,789
No. confirmed measles cases	387	429	780	543	219	44	190	1,824	2,248	6,664
Lab-confirmed	382	407	780	468	213	44	188	1,077	1,209	4,768
Confirmed by epi-link	2	10	-	73	-	-	1	747	1,039	1,872
Clinically compatible	3	12	-	2	6	-	1	-	-	24
No. lab-confirmed rubella cases	61	122	205	244	231	101	199	291	452	1,906
Districts with ≥1 specimen (%)^b^	20	43	56	56	48	41	46	57	65	-
Non-measles febrile rash per 100,000^c^	0.4	1.3	2.0	2.0	1.4	0.8	1.7	1.8	2.3	-

Abbreviations: No.=number, IDSR=Integrated Disease Surveillance and Response, epi-link=epidemiological link, MOPH=Ministry of Public Health

a Calculated by dividing the number suspected measles cases reported annually by the estimated annual population from MOPH and multiplying by 1,000,000; target: <5 per 1,000,000

b Calculated by dividing the number of districts reporting ≥1 suspected measles case with a blood specimen collected in a year by the number of districts expected to report and multiplying by 100; target: ≥80%

c Calculated by dividing the number of suspected measles cases reported annually that were discarded as non-measles due to a negative measles IgM test result by the annual estimated population from MOPH and multiplying by 100,000; target: ≥2 per 100,000

### Epidemiological description of the measles resurgence

**Figure 2:**
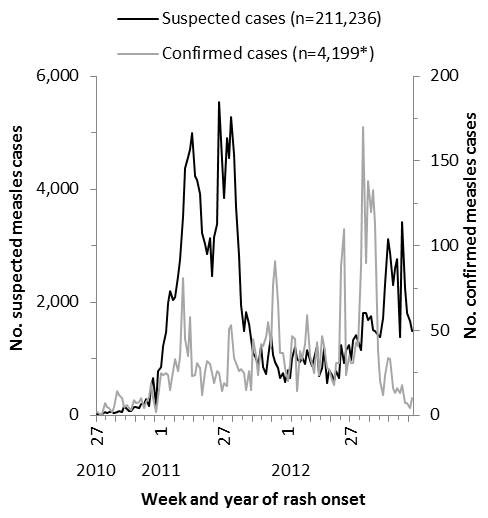
Suspected measles cases reported through Integrated Disease Surveillance and Response and confirmed measles cases reported through case-based surveillance by week, Democratic Republic of the Congo, July 1, 2010-December 31, 2012 (+53 cases confirmed by epidemiologic-link and reported through outbreak line-lists were missing date of rash onset)

During July 1, 2010-December 31, 2012, 211,236 suspected measles cases were reported through IDSR and 8,142 were reported through case-based surveillance. Of those reported through case-based surveillance, 4,252 (52%) were confirmed as measles (2,465 by laboratory testing and 1,787 by epidemiological link) and 866 (11%) were laboratory-confirmed as rubella (Figure 2 and Table 4). Of the 4,250 confirmed measles cases with age information, 2,839 (67%) were among children aged <5 years, and 879 (21%) were among children aged 5-9 years; 1,036 (24%) were among unvaccinated persons, and 2,168 (51%) were among persons with missing or unknown vaccination status (Table 5).

**Table 5:** Confirmed measles cases+ reported through measles case-based surveillance by age group and measles vaccination status, Democratic Republic of the Congo, July 1, 2010–December 31, 2012

	Cases with ≥1 dose MCV		Cases not vaccinated		Cases with missing or unknown vaccination status		Total cases	
Age group	No.	%	No.	%	No.	%	No.	%
<9 months	39	3.7	151	53.9	90	32.1	280	6.6
9–59 months	706	27.6	642	25.1	1,211	47.3	2,559	60.2
5–9 years	212	24.1	149	17.0	518	58.9	879	20.7
10–14 years	53	22.2	37	15.5	149	62.3	239	5.6
≥15 years	36	12.3	57	19.5	200	68.3	293	6.9
Total	1,046	24.6	1,036	24.4	2,168	51.0	4,250	100.0

Abbreviations: MCV=measles-containing vaccine, No.=number +Confirmed cases were defined as all laboratory-confirmed, epidemiologically-linked, and clinically-compatible measles cases

**Figure 3:**
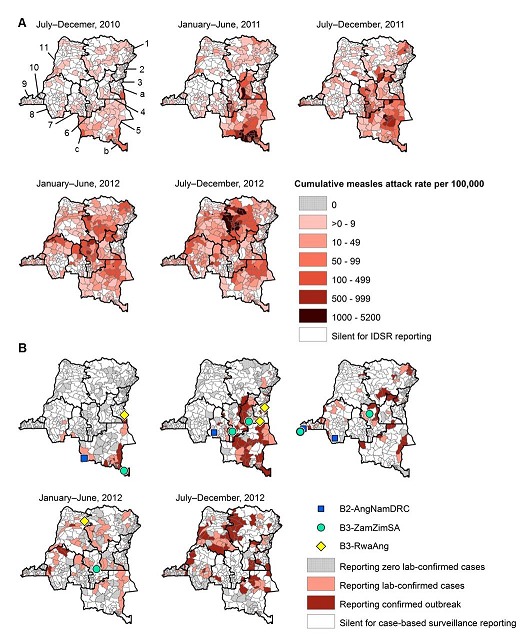
District maps of (A) cumulative measles attack rates reported through Integrated Disease Surveillance and Response and (B) confirmed measles outbreaks and measles virus strains reported through case-based surveillance for 6-month periods, Democratic Republic of the Congo, July 1, 2010-December 31, 2012 (provinces: 1. Province Orientale, 2. Nord Kivu, 3. Sud Kivu, 4. Maniema, 5. Katanga, 6. Kasaï Oriental, 7. Kasaï Occidental, 8. Bandundu, 9. Bas-Congo, 10. Kinshasa, and 11. Equateur; districts with first confirmed outbreaks: a. Lemera, b. Sakania, and c. Dilolo; each dot represents 1-8 viruses with the same genotype in a district within a month)

The first confirmed measles outbreaks of 2010 were reported in three geographic foci: 1) Lemera district bordering Burundi in Sud Kivu province in July 2010; 2) Sakania district bordering Zambia in Katanga province in August 2010; and 3) Dilolo district bordering Angola in Katanga province in September 2010. During July-December 2010, Sud Kivu and Katanga reported elevated cumulative measles attack rates and had 14 districts with confirmed outbreaks. In 2011, the epidemic peaked with confirmed measles outbreaks reported from 108 districts in all 11 provinces and the highest cumulative attack rates reported in Sud Kivu, Katanga, Maniema, and Kasaï Oriental provinces. In 2012, a second epidemic peak occurred with confirmed outbreaks reported from 126 districts in all 11 provinces and the highest cumulative attack rates reported from Province Orientale and Equateur (Figure 2, Figure 3 A and Figure 3 B).

**Figure 4:**
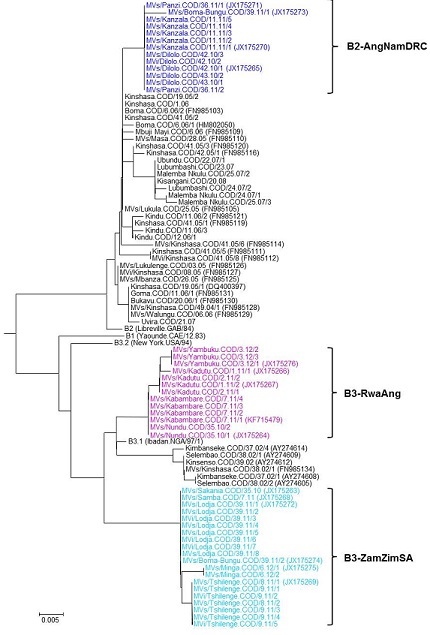
Phylogenetic analysis of the viral nucleoprotein gene of measles virus strains detected in the Democratic Republic of the Congo, July 1, 2010-February 28, 2012 (scale bar distance of 0.005=2 nucleotides substitutions in the 450 nucleotide sequenced region; sequence names contain information on the sequence source, district, country, epidemiologic week, year, and sequential case number; Genbank accession numbers indicated in brackets; abbreviations: MVi, measles virus sequenced from isolates; MVs, measles virus sequenced from clinical specimens; COD, Democratic Republic of the Congo; GAB, Gabon; NGA, Nigeria; CAE, Cameroon)

Measles virus genotype results were available from 47 confirmed measles cases in 13 (6%) of 218 districts that had a confirmed outbreak and 8 (73%) of 11 provinces. Three genetic groups of measles virus strains were detected each, associated with one of the three initial outbreak foci and thereafter clustered geographically. Of 47 measles viruses, 13 (28%) were from a group of B3.1 genotypic strains (B3-RwaAng) related to measles virus previously detected in Rwanda and Angola; these strains were first detected in a Sud Kivu district bordering Burundi and localized to northeastern DRC during the outbreak. Another 20 (43%) were from a group of B3.1 genotypic strains (B3-ZamZimSA) related or identical to measles viruses detected during 2009-2011 in Zambia, Zimbabwe and South Africa [31, 32]; these strains were first detected in a Katanga district bordering Zambia and localized to southern and central DRC. Finally, 13 (28%) were from a group of B2 genotypic strains (B2-AngNamDRC) related to those previously detected in Angola, Namibia, and DRC [13]; these strains were first detected in a Katanga district bordering Angola and localized to southern DRC (Figure 3 B, and Figure 4). One district in Bas-Congo province reported both B3-ZamZimSA and B2-AngNamDRC strains in the third-quarter of 2011 (Figure 3 B).

During the resurgence period, the national cumulative attack rate based on IDSR reporting was 270 cases per 100,000 population. The provinces with high cumulative attack rates (>100 cases per 100,000 population) were Katanga, Maniema, Kasaï Oriental, Province Orientale, Equateur, and Sud Kivu. Among these six provinces, estimated MCV1 coverage in the 2010 survey ranged from 46% to 66% (median: 54%), and four provinces had planned SIAs in 2010 that were not implemented. By contrast, estimated MCV1 coverage in the 2010 survey among the five provinces without high cumulative attack rates ranged from 49% to 91% (median 80%), and one province had a planned SIA in 2010 that was not implemented (Table 1 and Figure 1).

## Discussion

Following accelerated measles control efforts and dramatic decrease in measles incidence, a massive measles resurgence occurred in DRC. The results of this study suggest the resurgence was likely caused by an accumulation of measles-susceptible children not vaccinated due to low RI coverage and suboptimal SIA implementation. Furthermore, detailed epidemiological analyses necessary for guiding timely outbreak response were limited by under-performing case-based surveillance.

Despite implementation of the RED strategy and increasing MCV1 coverage in DRC during 2004-2012, coverage remained far below WHO-recommended targets for monitoring progress toward measles elimination [9, 33]. Results from coverage surveys indicated challenges with the reliability of administrative coverage and highlighted the need to conduct surveys to monitor coverage. In countries where only a single routine dose of measles vaccine is provided, achieving high coverage through both RI and SIAs is necessary to reduce measles incidence [22].

After implementation of the initial measles catch-up SIA during 2002-2006, measles incidence in DRC decreased dramatically. However, the catch-up SIA and follow-up SIAs were implemented in a phased manner to accommodate logistical challenges and resource limitations. This phased approach to implementation left a gap of 4-5 years between SIAs in some provinces, as well as a missed birth cohort in one province, which likely allowed for sustained measles virus transmission, similar to what has recently occurred in other large AFR countries [9, 34–36].

In DRC, widespread measles outbreaks continued during 2013-2014 among children who were targeted by SIAs and ORIs during 2011-2012. During 2013, 88,382 suspected and 1,321 confirmed cases were reported nationally, mostly from Province Orientale, Equateur and Kasaï Occidental; during 2014, 35,835 suspected and 1,643 confirmed cases were reported nationally (DRC MOPH and WHO-DRC unpublished data). Although administrative coverage from the 2011-2012 campaigns was generally high (>92%), suboptimal coverage was estimated by surveys conducted after the 2012 ORI in Katanga (68%), and in districts with outbreaks after the 2011 SIAs in Equateur and Province Orientale (<50%) [11, 17]. Additionally, some SIAs and ORIs were delayed because of the amount of time it took to secure and disburse funds, and to complete the extensive planning and logistics needed to reach poorly-accessible areas.

Although measles case-based surveillance with laboratory testing has been functional in DRC since 2003, performance indicators were unmet during 2004-2012, and challenges occurred with missing data (e.g. vaccination status). Surveillance guidelines specify that all suspected measles cases should be reported through measles case-based surveillance; however, the number of suspected measles cases reported through case-based surveillance was <4% of IDSR-reported cases [28]. This disparity was partly caused by the underutilization of measles case-based guidelines for the use of “line-lists” and the “epidemiologically-linked” case classification during confirmed outbreaks. These guidelines ensure that key epidemiological data, including age, vaccination status, and date of rash onset are collected for all cases, without requiring blood specimen collection and testing of all outbreak cases that might overburden the laboratory [29]. Case-based measles surveillance is a cornerstone of the measles elimination strategy; rapid case detection and laboratory confirmation is essential for rapid response, including effectively defining the target geographical area and age-group for SIAs and ORIs [37].

During 2009-2010, the African region experienced a measles resurgence with 28 of 46 AFR countries reporting laboratory-confirmed measles outbreaks, including large outbreaks in Burkina Faso, DRC, Ethiopia, Malawi, Nigeria, South Africa, Zambia, and Zimbabwe [9, 31, 32, 38, 39]. The regional resurgence was generally characterized by outbreaks with cases occurring among older children and young adults as a result of long-standing gaps in vaccination, combined with reduced risk of measles virus exposure due to decreased incidence since 2000 [9]. By contrast, 87% of measles cases during the resurgence in DRC occurred among children <10 years old, indicating persistent low population immunity among young children and on-going measles virus transmission. Virologic surveillance detected concurrent transmission of three separate measles virus strains in DRC; these strains were previously identified in neighboring countries, including a B3 strain detected in outbreaks throughout southern Africa during 2009-2011 [32].

During July 1, 2010-December 30, 2012, high measles attack rates occurred in provinces (Katanga, Maniema, Kasaï Oriental, Province Orientale, Equateur, and Sud Kivu) that had estimated MCV1 coverage in the 2010 survey lower than the national estimate (67%) and did not implement planned 2010 SIAs. Kasaï Occidental province had a relatively low attack rate (68 cases per 100,000 population) during 2010-2012 despite having 49% estimated MCV1 coverage in the 2010 survey and not implementing a planned 2010 SIA. The lower reported attack rate in Kasaï Occidental might have reflected underreporting from poor surveillance quality or a relative delay in the progression of the outbreak due to the northwest geographic spread of the outbreak over time from the initial foci in the south and east; in 2013, the measles attack rate in Kasaï Occidental increased to >100 cases per 100,000 population (DRC MOPH and WHO-DRC unpublished data). An additional factor that may have impacted relative attack rates among provinces during the measles outbreak was the increase in number of IDPs and refugees, as well as affected areas, during 2010-2013 [18–21]. There was some overlap between the provinces with high measles attack rates and those with large numbers of displaced persons; however, a detailed analysis of the temporal and geographical relationship of these events was not performed.

The study analyses had limitations. First, reported administrative coverage was likely overestimated due to unreliable denominators, as indicated by coverage >100% and lower coverage estimates by survey. Second, analysis of campaign quality was limited in part by the small number of coverage surveys conducted and lack of detailed post-campaign reports available. Not all available coverage surveys were performed using probability-based sampling methods, so representativeness of the estimates could not be determined. Third, under-reporting through case-based surveillance and the number of districts that were silent for IDSR reporting suggest measles incidence was likely underestimated. Fourth, because suspected measles cases reported through IDSR were not confirmed, some illnesses attributable to other causes of febrile rash, including rubella, were likely misclassified in IDSR as suspected measles.

## Conclusion

To achieve measles elimination in DRC, we recommended specific efforts to improve case-based surveillance and increase two-dose measles vaccination coverage. Realistic plans need to be developed to fully implement case-based surveillance, including adequate costing and resource mobilization for blood collection supplies, testing, and specimen transport. Periodic refresher trainings should be provided to ensure the implementation of measles case-based surveillance according to WHO guidelines [22, 29]. Additionally, WHO-recommended measles case-based surveillance performance indicators should be calculated monthly in a surveillance bulletin provided to all districts and used as a tool for supervision, management, and surveillance strengthening [29]. Measures to strengthen RI services are needed, including increased supportive supervision, human resources, and training, as well as improved micro-planning, vaccine management, and logistics. Because of suboptimal MCV1 coverage, periodic high-quality, nationwide SIAs should be implemented, ideally without phases and covering the entire country during a 4-7 day period; however, if a limitation in resources necessitates a phased approach, then the number of phases and the time needed to cover the entire country should be minimized [40]. SIA frequency and target age groups should be based on epidemiological analyses, and adequate resources made available to ensure optimal implementation [41]. According to WHO guidelines, SIA planning should start at least 6-8 months in advance, with close supervision and monitoring during implementation, and a post-campaign coverage survey [40]. To ensure timely outbreak response, a national measles outbreak preparedness plan is needed [37].

During September 2013-August 2014, with funding and support from Gavi the Vaccine Alliance, WHO, UNICEF, and the Measles & Rubella Initiative, the DRC MOPH conducted a phased nationwide measles SIA targeting children aged 6 months-9 years, incorporating SIA best practices identified during a workshop in Kinshasa in April 2013, and post-campaign coverage surveys were completed in four provinces. The country will need to conduct the next follow-up SIA in 2016 and should begin the planning process in early 2015. To achieve measles elimination in AFR by 2020, additional commitments and resources will be needed to implement strategies for increasing two-dose vaccination coverage and improving surveillance in DRC.
